# Gene expression profiling and pathway analysis data in MCF-7 and MDA-MB-231 human breast cancer cell lines treated with dioscin

**DOI:** 10.1016/j.dib.2016.05.040

**Published:** 2016-05-25

**Authors:** Pranapda Aumsuwan, Shabana I. Khan, Ikhlas A. Khan, Larry A. Walker, Asok K. Dasmahapatra

**Affiliations:** a National Center for Natural Product Research, University of Mississippi, MS 38677, USA; b Division of Pharmacology, Department of BioMolecular Sciences, University of Mississippi, MS 38677, USA; c Division of Pharmacognosy, Department of BioMolecular sciences, School of Pharmacy, University of Mississippi, MS 38677, USA; d University of Mississippi Cancer Institute (Oxford Campus), School of Pharmacy, University of Mississippi, MS 38677, USA

## Abstract

Microarray technology (Human OneArray microarray, phylanxbiotech.com) was used to compare gene expression profiles of non-invasive MCF-7 and invasive MDA-MB-231 breast cancer cells exposed to dioscin (DS), a steroidal saponin isolated from the roots of wild yam, (*Dioscorea villosa*). Initially the differential expression of genes (DEG) was identified which was followed by pathway enrichment analysis (PEA). Of the genes queried on OneArray, we identified 4641 DEG changed between MCF-7 and MDA-MB-231 cells (vehicle-treated) with cut-off log2 |fold change|≧1. Among these genes, 2439 genes were upregulated and 2002 were downregulated. DS exposure (2.30 μM, 72 h) to these cells identified 801 (MCF-7) and 96 (MDA-MB-231) DEG that showed significant difference when compared with the untreated cells (*p*<0.05). Within these gene sets, DS was able to upregulate 395 genes and downregulate 406 genes in MCF-7 and upregulate 36 and downregulate 60 genes in MDA-MB-231 cells. Further comparison of DEG between MCF-7 and MDA-MB-231 cells exposed to DS identified 3626 DEG of which 1700 were upregulated and 1926 were down-regulated. Regarding to PEA, 12 canonical pathways were significantly altered between these two cell lines. However, there was no alteration in any of these pathways in MCF-7 cells, while in MDA-MB-231 cells only MAPK pathway showed significant alteration. When PEA comparison was made on DS exposed cells, it was observed that only 2 pathways were significantly affected. Further, we identified the shared DEG, which were targeted by DS and overlapped in both MCF-7 and MDA-MB-231 cells, by intersection analysis (Venn diagram). We found that 7 DEG were overlapped of which six are reported in the database. This data highlight the diverse gene networks and pathways in MCF-7 and MDA-MB-231 human breast cancer cell lines treated with dioscin.

**Specification Table**

**Table:** 

Subject area	Biology
More specific subject area	Breast Cancer
Type of data	Table, Figure
How data was acquired	Microarray analysis; data were done by Phalanx Biotech Group using Human OneArray (array version HOA 6.1) which contains 31,741 mRNA probes that can detect 20, 672 genes in human genome.
Data format	Analyzed
Experimental factors	Both MCF-7 and MDA-MB-231cells (~500×10^3^ cells) were treated with DS (2.30 µM) for three days followed by RNA extraction and analysis.
Experimental features	MCF-7 and MDA-MB-231 cells were cultured in phenol red free DMEM-F12 (1:1) medium supplemented with 10% dextran charcoal treated fetal bovine serum, 50 U/mL penicillin and 50 µg/mL streptomycin as Pen-Strep and 2 mM of l-glutamine at 37 °C in a humidified atmosphere of 95% air and 5% CO_2_. The cells (~500×10^3^ cells) were allowed to attach in the 25 cm^3^ culture flasks in 6 mL volume and after 24 h the cultures were treated with DS (2. 30 µM) for three days.
Data source location	N/A
Data accessibility	Data is within this article and available at the NCBI database via GEO series accession numbers GEO: GSE79465; GEO: GPL 19137; GEO:GSM2095708; GEO:GSM2095709; GEO:GSM2095710

**Value of the data**

• May stimulate further research on the utility of DS as a preventive agent of metastatic breast cancer.
• May facilitate new therapies to target specific genes that are associated with metastatic breast cancer.
• Genes participating in MAPK signaling pathways are the probable targets of breast cancer metastasis.

## 1 Data

**Fig. 1:**
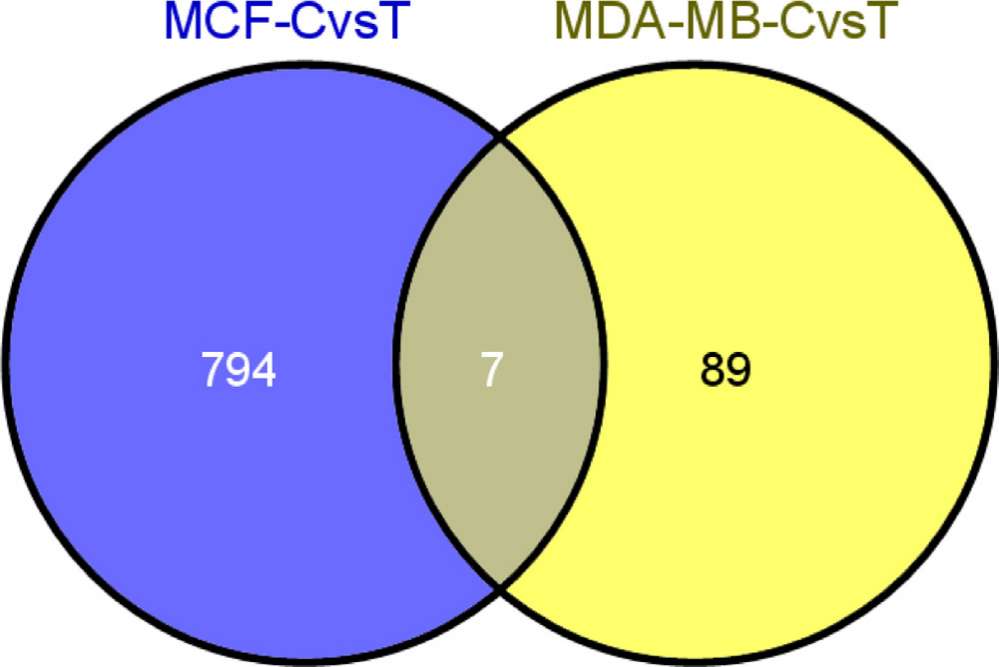
Venn diagram of the overlap among DEGs of MCF-7 and MDA-MB-231 cells exposed to DS (2.30 µM, 72 h). The MCF-7 and MDA-MB-231 cells shared seven genes of which six genes were found in the data base.

Table 1 showed data on the global gene expression profile in MCF-7 and MDA-MB-231 cell lines treated with vehicle (DMSO) or DS in vitro. Table 2, Table 3, Table 4 showed gene ontology analysis based on molecular functions (Table 2), biological processes (Table 3), and cellular components (Table 4). Various canonical pathways, which were significantly altered between the cell lines (vehicle-treated) or after DS treatment, were presented in Table 5. The genes that were overlapped between these two cell lines (MCF-7 and MDA-MB-231) after DS treatment were listed in Table 6 and in a Venn diagram format in Fig. 1.

## 2 Experimental design, materials and methods

### 2.1 Cell culture, DS treatment, and extraction of nucleic acids

The detailed procedure of cell culture, treatment with DS, and the isolation of RNA have been described in our previous study [1]. In brief, human breast adenocarcinoma, MCF-7 (ER^+^) and MDA-MB-231 (ER^−^) cells were maintained in phenol red free DMEM-F12 (1:1) medium supplemented with 10% dextran charcoal treated fetal bovine serum, 50 U/mL penicillin and 50 µg/mL streptomycin and 2 mM of l-glutamine. The cells (~500×10^3^ cells) were allowed to attach in the 25 cm^3^ culture flasks in 6 mL volume for 24 h before treating with DS (2.30 µM) for three days. After complete removal of the media, the cells were trypsinized, resuspended in the medium, and washed twice with PBS. RNA extraction was made by Trizol reagent as described previously [1]. Briefly, Trizol reagent (Invitrogen, Carlsberg, CA) was used to lyse the cells. Chloroform was added to the lysate for phase separation. The clean aqueous phase (RNA) was transferred to a clean 1.5 ml Eppendorf tube and RNA was precipitated by 2-propanol. After a quick wash in 75% ethanol, the extracted RNA was dissolved in nuclease-free water. The samples (extracted RNA) were further treated with DNase I (Promega, Madison, WI), to remove DNA contamination, if any. Finally, the concentration of RNA was determined by NanoDrop 2000c (Thermo Fisher Scientific, Waltham, MA) and the samples were stored at −80 °C until sending to Phalanx Biotech Group for microarray analysis.

### 2.2 Microarray analysis

Microarray analysis was carried out by Phalanx Biotech Group using OneArray (array version HOA 6.1) which contains 31,741 mRNA probes that can detect 20, 672 genes in human genome. In brief, the purity of the extracted RNA was checked using NanoDrop ND-1000. The Pass criteria for absorbance ratios are established as A260/A280≥1.8 and A260/A230≥1.5. RIN values are ascertained using Agilent RNA 6000 Nano assay to determine RNA integrity. Pass criteria for RIN value is established at >6. Genomic DNA (gDNA) contamination was evaluated by gel electrophoresis. Any RNA that did not meet these criteria was excluded from the analysis.

Target preparation was performed using an Eberwine-based amplification method with Amino Allyl MessageAmp II aRNA Amplification Kit (Ambion, AM1753) to generate amino-allyl antisense RNA (aa-aRNA). Labeled aRNA coupled with NHS-CyDye (Cy5) was prepared and purified prior to hybridization. Purified coupled aRNA was quantified using NanoDrop ND-1000; pass criteria for CyDye incorporation efficiency at >15 dye molecular/1000 nt. All the raw data are available in NCBI׳s gene expression Omnibus and are accessible through GEO series accession number GSE79465 (http://www.ncbi.nlm.nih.gov/geo/query/acc.cgi?acc=GSE79465).

### 2.3 Gene expression data analysis

Global scaling normalization (scatter plot, histogram and volcano plot, principal component analysis) was carried out, and the fold changes (cut-off (log2 |fold change|≧1)) were calculated based on the relative signal intensities (scanned by Agilent 0.1 XDR protocol). A filtering step was performed using Rosetta error model [2] which allowed for determination of the statistical significance of every pair wise gene between different groups. The default multiple testing corrections used was Benjamini and Hochberg [3] false discovery rate with a *q* value cutoff <0.05. The testing correction was the least stringent of all corrections and provided a good balance between the discovery of statistically significant genes and the limitation of false positive occurrences by removing all gene spots with a *q* value >0.05 in all conditions. This procedure narrowed the list of genes to those significantly affected by DS treatment. Gene annotation was based on two data bases: NCBI ref seq release 57.ensembl release 70 cDNA sequences and homo_sapiens_core_70_37. Finally the pathway enrichment analysis (PEA) was utilized to group and display genes with similar expression profiles. The online tool Database for Annotation, Visualization, and Integrated Discovery (DAVID) [4] was used for PEA. The selected KEGG (Kyoto Encyclopedia of Genes and Genomes) pathways with an adjusted EASE (Expression Analysis Systematic Explore) score *p* value ≤0.05 and count >2. Data gained by this technique may help to understand more on in vitro studies of botanical natural products used in breast cancer treatment. The pathway analysis was used to examine functional correlations within the cell lines and different treatment groups. Data sets containing gene identifiers and corresponding expression values were uploaded into the application. Each gene identifier was mapped to its corresponding gene object in the KEGG pathway map with an adjusted EASE (Expression Analysis Systematic Explore) score *p* value ≤0.05 and count >2. Networks were “named” on the most common functional group(s) present in the database. Canonical pathway analysis (GeneGo maps) as evaluated acknowledged function-specific genes significantly present within the network [5].

## Figures and Tables

**Table 1:** Number of differentially expressed genes in MCF-7 and MDA-MB-231 cells.

	Comparison	Up-regulated (number)	Down-regulated (number)
1	MCF-7C/MDA-MB-231C	2439	2002
2	MCF-7C/MCF-7T	395	406
3	MDA-MB-231C/MDA-MB-231T	36	60
4	MCF-7T/MDA-MB-231T	1700	1926

**Table 2:** Gene ontology analysis based on molecular functions.

Gene set name	Number of genes in the gene set	Number of genes overlap			
		MCF-7 (T/C)	MDA-MB-231 (T/C)	MCF-7 (C)/ MDA-MB-231 (C)	MCF-7 (T)/MDA-MB-231 (T)
Magnesium ion binding	452	38⁎	–	125⁎	97
Cytokine activity	195	–	8⁎	–	–
Enzyme binding	523	38	–	141⁎	109
Actin binding	326	23	–	95⁎	76
Cytoskeletal protein binding	504	–	–	135⁎	102
Purine ribonucleotide binding	1836	95	–	410⁎	306
Ribonucleotide binding	1836	95	–	410⁎	–
Purine nucleotide binding	1918	96	–	424⁎	323
Nucleotide binding	2245	110	–	485⁎	–
Adenyl ribonucleotide binding	1497	81	–	332⁎	–
ATP binding	1477	81		328⁎	251
Protein domain specific binding	331	–	–	89⁎	–
Nucleoside binding	1612	84	–	353⁎	278
Purine nucleoside binding	1601	83	–	350⁎	273
Adenyl nucleotide binding	1577	82	–	345⁎	270
Transcription factor binding	513	29	–	127⁎	–
Enzyme activator activity	335	21	–	88⁎	62

⁎ The asterisk indicates *q*<0.05 [3].

**Table 3:** Gene ontology analysis based on biological process.

Gene set name	Number of genes in the gene set	Number of genes in overlap			
		MCF-7 (T/C)	MDA-MB-231 (T/C)	MCF-7 (C)/ MDA-MB-231 (C)	MCF-7 (T)/MDA-MB-231 (T)
Protein complex biogenesis	505	47⁎	–	129⁎	–
Protein complex assembly	505	47⁎	–	129⁎	–
Macromolecular complex assembly	665	55⁎	–	–	–
Macromolecular complex subunit organization	710	56⁎	–	165	–
Protein oligomerization	174	20⁎	–	50	–
Protein amino acid phosphorylation	667	47⁎	–	156	–
Protein heterooligomerization	52	10⁎	–	17	–
Negative regulation of cell proliferation	361	22	10⁎	81	86⁎
Cell cycle	776	46	–	210⁎	–
Regulation of cell death	815	52	11	205⁎	165⁎
Regulation of apoptosis	804	52	11	202⁎	163⁎
Induction of programmed cell death	321	21	–	94⁎	73⁎
Regulation of programmed cell death	812	52	11	202⁎	163⁎
Induction of apoptosis	320	21	–	93⁎	72⁎
Positive regulation of cell death	435	27	–	119⁎	95⁎
Cell cycle process	565	34	–	147⁎	–
Regulation of binding	153	78	4	52⁎	42⁎
Positive regulation of Programmed cell death	433	27	–	117⁎	94⁎
Positive regulation of apoptosis	430	27	–	116⁎	93⁎
Cell death	719	47	8	176⁎	146⁎
Mitotic cell cycle	370	–	–	100⁎	–
Cell division	295	–	–	83⁎	–
Death	724	47	8	176⁎	–
Programmed cell death	611	40	8	152⁎	–
Apoptosis	602	38	8	150⁎	–
Regulation of DNA binding	121	–	4	41⁎	35⁎
Regulation of cell proliferation	787	48	12	182	183⁎
Positive regulation of cell proliferation	414	29	–	–	97⁎
Cell proliferation	436	28	–	110	99⁎
Neuron differentiation	438	–	–	–	98⁎
Death	724	47	8	–	146⁎
Regulation of locomotion	192	15	–	56	50⁎
Cell migration	276	–	5	69	66⁎
Regulation of cell motion	193	16	–	56	50⁎
Blood vessel development	245	–	–	64	60⁎
Neuron projection development	256	–	–	–	62⁎
Vasculature development	251	–	–	64	61⁎
Cell projection organization	368	–	–	91	82⁎
Regulation of cellular component size	271	–	6	66	64⁎
Transmembrane receptor protein serine/threonine kinase signaling pathway	103	12	–	35	31⁎
Regulation of cell migration	169	14	–	51	44⁎
Hemopoietic or lymphoid organ development	260	–	–	60	61⁎
Positive regulation of developmental process	278	18	6	72	64⁎
Axon guidance	107	–	–	–	31⁎
Hemopoiesis	236	–	–	–	56⁎
Positive regulation of locomotion	98	12	–	32	29⁎
Locomotory behavior	274	–		–	63⁎
Response to vitamin	66	–		–	22⁎

⁎ The asterisk indicates *q*<0.05 [3].

**Table 4:** Gene ontology analysis based on cellular component.

Gene set name	Number of genes in the gene set	Number of genes in overlap			
		MCF-7 (T/C)	MDA-MB-231 (T/C)	MCF-7 (C)/ MDA-MB-231 (C)	MCF-7 (T)/MDA-MB-231 (T)
Membrane-enclosed Lumen	1856	111⁎	–	397⁎	–
Organelle lumen	1820	108⁎	–	391⁎	300
Intracellular organelle Lumen	1779	106⁎	–	382⁎	291
Nuclear lumen	1450	91⁎	–	312⁎	243
Nucleoplasm	882	62⁎	–	186	–
Intracellular Non-membrane-bounded Organelle	2596	134⁎	–	–	–
Non-membrane-bounded Organelle	2596	134⁎	–	–	–
Cytosol	1330	74⁎	–	285⁎	–
Cytoskeleton	1381	74⁎	–	–	–
Nuclear matrix	56	9⁎	–	–	–
Nuclear periphery	61	9⁎	–	–	–
Extracellular space	685	–	12⁎	–	
Extracellular region part	960	–	14⁎	–	–
Lytic vacuole	211	17	–	71⁎	56⁎
Lysosome	211	–	–	71⁎	56⁎
Vacuole	252	18	–	79⁎	62⁎
Basolateral plasma Membrane	203	14	–	64⁎	–
Non-membrane-bounded Organelle	2596	134	–	543⁎	–
Intracellular Non-membrane-bounded Organelle	2596	134	–	543⁎	413⁎
Anchoring junction	172	14	–	52⁎	46⁎
Adherens junction	155	–	–	48⁎	41⁎
Golgi apparatus	872	–	–	197⁎	150
Mitochondrion	1087	56	–	239⁎	–
Cell fraction	1083	–	–	237⁎	209⁎
Nucleolus	698	–	–	107⁎	129
Cell leading edge	138	–	–	41⁎	37⁎
Extracellular matrix	345	–	5	–	78⁎
Insoluble fraction	839	–	–	–	159⁎

⁎ The asterisk indicates *q*<0.05 [3].

**Table 5:** Gene set enrichment analysis based on the canonical pathway.

Gene set name	Number of genes in the gene set	Number of genes in overlap			
		MCF-7 (T/C)	MDA-MB-231 (T/C)	MCF-7 (C)/ MDA-MB-231 (C)	MCF-7 (T)/MDA-MB-231 (T)
MAPK signaling pathway	267	–	7⁎	70	56
Pathways in cancer	328	27	–	99⁎	76⁎
Apoptosis	87	–	–	34⁎	23
Lysosome	117	–	–	41⁎	37⁎
VEGF signaling pathway	75	–	–	29⁎	–
Focal adhesion	201	–	–	60⁎	–
Prostate cancer	89	–	–	32⁎	–
mTOR signaling pathway	52	–	–	21⁎	–
Pancreatic cancer	72	–	–	26⁎	–
Colorectal cancer	84	–	–	29⁎	–
Renal cell carcinoma	70	–	–	25⁎	–
Regulation of actin cytoskeleton	215	16	–	59⁎	–
Small cell lung cancer	84	–	–	28⁎	–

⁎ The asterisk indicates *q*<0.05 [3].

**Table 6:** List of genes overlapped between the two cell lines.

Gene symbol	Description of the gene	Log2 (ratio)			
		MDA-MB-231C/MCF-7C	MCF-7T/MCF-7C	MDA-MB-231T/MDA-MB-231C	MDA-MB-231T/MCF-7T
ERRFI1	ERBB receptor feedback inhibitor 1	0.01	1.33	1.06	−0.35
MMP1	Matrix metallopeptidase 1 (interstitial collagenase)	1.59	2.70	2.09	0.96
SOD2	Superoxide dismutase 2, mitochondrial	2.54	1.04	1.08	2.61
IL24	Interleukin 24	−0.93	1.44	2.86	0.37
PTRF	Polymerase I and transcript release factor	−1.54	−2.35	−1.03	−0.23
ALKBH5	AlkB, alkylation repair homolog 5 (*E. coli*)	−0.70	−1.36	−1.01	−0.40
